# Global Antiphospholipid Syndrome Score (GAPSS) in Patients with Systemic Lupus Erythematosus

**DOI:** 10.1134/S160767292370028X

**Published:** 2023-10-13

**Authors:** F. A. Cheldieva, T. M. Reshetnyak, A. A. Shumilova, K. S. Nurbaeva, M. V. Cherkasova, A. M. Lila, E. L. Nasonov

**Affiliations:** 1 Laboratory of Thromboinflammation, Nasonova Research Institute of Rheumatology, Moscow, Russia; 2 Department of Rheumatology, Russian Medical Academy of Continuous Professional Education of the Ministry of Health of the Russian Federation, Moscow, Russia; 3 Sechenov First Moscow State Medical University, Ministry of Health of the Russian Federation (Sechenov University), Moscow, Russia

**Keywords:** disease activity, antiphospholipid syndrome, systemic lupus erythematosus, antiphospholipid antibodies, thrombosis, obstetric pathology

## Abstract

The Global Antiphospholipid Syndrome Score (GAPSS) is a tool proposed to quantify the risk of clinical manifestations associated with antiphospholipid antibodies (aPL) and certain cardiovascular risk factors. To validate GAPSS in a cohort of patients with systemic lupus erythematosus in Russia. 115 patients with SLE were included in the study, including 51 (44%) patients with systemic lupus erythematosus (SLE) with antiphospholipid syndrome (APS), 14 (12%) SLE patients with aPL, and 50 (44%) patients with SLE. There was a history of thrombosis in 58 (50%) out of 115 patients; of them, 14 (24%) had arterial thrombosis, 29 (50%) had venous thrombosis, and 15 (26%) had combined thrombosis. Pregnancy against the background of the disease occurred in 43 women included in the study. Of them, 29 (67%) had obstetric pathology. Patients with thrombosis and obstetric pathology had a GAPSS score of 7.17 ± 5.64 versus 4.48 ± 4.55 without these manifestations (*p* = 0.0003). There was a significant association between GAPSS levels and thrombosis: patients with thrombosis had a GAPSS of 7.31 ± 5.70, those without thrombosis—4.00 ± 4.81 (*p* = 0.001). GAPPS values were higher in arterial thrombosis compared to venous thrombosis (10.40 ± 25.30 versus 5.82 ± 5.28, *p* = 0.01). GAPSS levels  ≥ 6 and  ≥10 were analyzed to select GAPSS values at which a high risk of recurrent thrombosis and/or obstetric pathology could be indicated. All GAPSS levels had a significant association with clinical manifestations of APS. The quality of GAPSS by ROC analysis showed an area under the curve (AUC) for GAPSS of 0.697. GAPSS can be used to assess the risk of recurrence or development of thrombosis and/or obstetric pathology in patients with SLE in the Russian Federation. The GAPSS ≥6 values should be used to stratify patients with SLE into high risk group for recurrence of vascular complications. Further prospective follow-up is needed to confirm the value of GAPSS.

## INTRODUCTION

Antiphospholipid syndrome (APS) is an acquired thrombophilic condition, the main clinical manifestations of which are vascular thrombosis of any localization and caliber and obstetric pathology (recurrent fetal loss syndrome) [1]. Serological markers of APS are antiphospholipid antibodies (aPLs), which, according to international classification criteria, include IgG/IgM antibodies to cardiolipin (aCL), IgG/IgM antibodies to β_2_-glycoprotein 1 (anti-β_2_GP1) and lupus anticoagulant (LA) [2]. The long-term prognosis for patients with APS largely depends on the risk of recurrent thrombosis and the presence of other clinical manifestations and laboratory markers included in the number of extracriterion manifestations of the disease [3–5]. The latter include livedo reticularis, skin ulcers, thrombocytopenia, microangioencephalopathy, nephropathy (with the exclusion of other diseases), pathology of the valvular apparatus of the heart (Libman–Sacks non-infectious endocarditis), antibodies to the phosphatidylserine/prothrombin complex (aPs/Pt), antibodies to domain I of β_2_‑glycoprotein 1 (β_2_GP1), IgA aPL, antibodies to annexin, etc. [3, 6]. One of these signs may be the only manifestation in the debut that determines the tactics of treatment and the subsequent outcome of the disease.

According to Grika et al. [7], as the disease progressed, the prevalence of initial clinical signs of APS increased despite therapy. A number of studies showed that thrombotic manifestations of APS are the main predictors of irreversible organ damage and death in patients with SLE [8]. The progression of the disease largely depends on the profile and levels of aPL. Despite the existence of laboratory recommendations for the study of aPL, there are interlaboratory discrepancies in the evaluation of the results of their determination. The lack of standardization of diagnostic systems leads to heterogeneous results. Therefore, to date, the stratification of various aPLs by their type and levels has not been fully carried out. This problem can be solved by developing a quantitative method for assessing the risk of recurrence of clinical manifestations of APS. To date, there is no such universally accepted index for APS, despite the widespread use of various assessment indices in other rheumatic diseases.

In 2013, Sciascia et al. [9] developed Global AntiphosPholipid Syndrome Score (GAPSS), which combines the aPL profile and the conventional risk factors for cardiovascular complications. GAPSS makes it possible to assess the risk of developing clinical manifestations in APS patients taking into account the non-criteria signs of the disease, in particular, aPs/Pt. GAPSS is calculated for each patient as the sum of scores for various risk factors for thrombosis and cardiovascular events (Table 1). When GAPPS ≥ 10, the risk of thrombosis is considered high, the maximum value of the index is 20.

**Table 1.** Global AntiphosPholipid Syndrome Score (GAPSS)

Parameters	Points
IgG or IgM antibodies to cardiolipin	5
IgG or IgM antibodies to β_2_ glycoprotein 1	4
Lupus anticoagulant	4
IgG or IgM antibodies to phosphatidylserine-prothrombin complex	3
Hyperlipidemia	3
Arterial hypertension	1

IgG, immunoglobulin G; IgM, immunoglobulin M.

The necessity to assess the risk of APS complications using GAPSS was confirmed in prospective studies on a separate cohort of SLE patients [10] and in a group of patients with primary APS [11]. Other researchers pointed to the necessity to use GAPSS in APS as a potential tool to quantify the risk of recurrence of the clinical manifestations of APS [12, 13].

The aim of this study was to compare the informativeness of determining a high risk of recurrent thrombosis and/or obstetric pathology using the GAPSS index in the Russian cohort of patients with systemic lupus erythematosus (SLE) using GAPSS thresholds ≥6 and ≥10.

## MATERIALS AND METHODS

The study included 115 patients. Of  these, 50 (44%) had SLE, 51 (44%) had SLE with APS (SLE + APS), and 14 (12%) had SLE with aPL (SLE + aPL) (Table 2). The SLE patients without aPL had a shorter disease duration (*p* < 0.0001) and were younger than the SLE + APS patients (*p* = 0.003). The duration of disease and the age of patients in the SLE + aPL group were less (*p* = 0.0006 and *p* = 0.03, respectively) than in the SLE + APS group. In the SLE + APS patients, thrombosis was more common than in the SLE without aPL group (*p* < 0.0001). There were no statistically significant differences in the incidence of obstetric pathology in groups SLE + APS and SLE without aPL (*p* = 0.07).

**Table 2.** Characteristics of patients included in the study

Parameters		SLE with APS (*n* = 51)	SLE with aPL (*n* = 14)	SLE without aPL (*n* = 50)	Total (*n* = 115)
Average age, Мe [25; 75 percentiles], years		41.0 [34.0–47.0]	35.0 [20.0–37.0]*	30.5 [24.0–42.0]**	36.0 [27.0–45.0]
Duration of the disease, Мe [25; 75 percentiles], years		16.0 [8.0–20.0]	4.5 [2.0–7.0]***	4.0 [1.5–8.6]****	7.3 [2.5–17.0]
Sex: male/female, abs		40 (78)/11 (22)	14 (100)/0 (0)	42 (84)/8 (16)	96 (83)/19 (17)
History of thrombosis, abs (%)		44 (86)*****	0 (0)	14 (27)	58 (50.4)
Obstetric pathology ******, *n* (%)/*n*		22 (79)/28	0 (0)/0	7 (47)/15	29 (67)/43
аCL, *n* (%)	IgG	31 (61)	8 (57)	0 (0)	39 (34)
	IgM	8 (16)	6 (43)	0 (0)	14 (12)
	IgG + IgM	5 (10)	5 (36)	0 (0)	10 (9)
anti-β_2_-GP1, *n* (%)	IgG	36 (71)	9 (64)	0 (0)	45 (39)
	IgM	10 (20)	7 (50)	0 (0)	17 (15)
	IgG + IgM	9 (18)	6 (43)	0 (0)	15 (13)
aPS/PT, *n* (%)	IgG	25 (49)	6 (43)	3 (6)	34 (30)
	IgM	14 (27)	9 (64)	2 (4)	25 (22)
	IgG + IgM	9 (18)	6 (43)	0 (0)	15 (13)
Lupus anticoagulant*******, *n* (%)		8 (73)/11	4 (44)/9	0 (0)/15	12 (34)/35
Arterial hypertension, *n* (%)		27 (53)	4 (29)	14 (28)	45 (39)
Hyperlipidemia, *n* (%)		12 (24)	1 (7)	11 (22)	24 (21)
Therapy, *n* (%)	Anticoagulant	41 (80)	2 (14)	16 (32)	59 (51)
	Low-dose aspirin	18 (35)	5 (36)	12 (24)	35 (30)
	Hydroxychloroquine	46 (90)	10 (71)	46 (92)	102 (89)
	Glucocorticoid	42 (82)	8 (57)	47 (94)	97 (84)

SLE, systemic lupus erythematosus; APS, antiphospholipid syndrome; aPL, antiphospholipid antibodies; aCL, antibodies to cardiolipin; IgG, immunoglobulin G; IgM, immunoglobulin M; anti-β_2_-GP1, antibodies to beta-2 glycoprotein 1; аPS/PT, antibodies to phosphatidylserine-prothrombin complex; * *р* = 0.03 compared to SLE with APS; ** *р* = 0.003 compared to SLE with APS; *** *р* = 0.0006 compared to SLE with APS; **** *р* < 0.0001 compared to SLE with APS; ***** *р* < 0.0001 compared to SLE without aPL; ****** obstetric pathology was calculated in women who had pregnancy in their disease course; ******* numeral before the slash indicates the number of patients positive for lupus anticoagulant; after the slash, the total number of patients who were tested for lupus anticoagulant.

The diagnosis of SLE corresponded to the classification criteria of the Systemic Lupus International Collaborating Clinics (SLICC), 2012 [14]. The diagnosis of APS corresponded to the international classification criteria of 2006 [2]. SLE activity was assessed using the Systemic Lupus Erythematosus Disease Activity Index (SLEDAI) [15]. A score of 0 was taken for the absence of SLE activity, 1–5 points for low activity, 6–10 points for moderate activity, 11–19 points for high activity, and ≥20 points for very high activity. Organ damage was assessed by the damage index (DI) SLICC/ACR [16]. A score of 0 points was taken for no damage, 1 point for low DI, 2–4 points for medium DI, and more than 4 points for high DI.

The median of the SLEDAI index was 4.00 [2.00–11.00], and the median of SLICC PI was 0.00 [0.00–2.00]. In SLE + APS patients, the SLEDAI index was statistically significantly lower (4.00 [2.00–8.00] and 6.50 [2.00–16.00], respectively; *p* = 0.004), and PI was statistically significantly higher than in the patients with SLE without aPL (1.00 [0.00–3.00] and 0.00 [0.00–1.00], respectively; *p* = 0.005).

All patients included in the study were examined and received the main therapy in inpatient or outpatient units of Nasonova Research Institute of Rheumatology. The frequency of therapy with GC and hydroxychloroquine in the groups of patients was comparable. Half of the patients (51%) received anticoagulants (mainly vitamin K antagonists), and the number of patients with anticoagulants in the SLE + APS group significantly predominated (Table 2). All patients underwent standard clinical, laboratory, and instrumental examinations prior to inclusion in the study and during follow-up.

The immunological study included the determination of antinuclear autoantibodies (ANA) using the hep-2 cell line, antibodies to double-stranded DNA (anti-dsDNA), C3 and C4 complement components, as well as antibodies to the cytoplasmic antigen SSA (Ro, anti-Ro/SSA), cytoplasmic antigen SSB (La, anti La/SSB), Sm antigen, LA, aCL, and anti-β_2_GP1.

IgG/IgM aCL and IgG/IgM anti-β_2_GP1 were determined by enzyme immunoassay (ELISA) on an Alegria automatic analyzer for laboratory diagnostics of autoimmune diseases (Orgentec Diagnostika GmbH, Germany) with a reagent kit for detection of antibodies from Orgentec Diagnostika GmbH (Germany). IgG aCL was measured in phospholipid-binding activity of IgG aCL per 1 µg/mL in GPL units, and IgM aCL was measured in phospholipid-binding activity of IgM aCL per 1 µg/mL in MPL. IgG/IgM anti-β_2_GP1 was measured in U/mL. Values >25.00 GPL for IgG aCL, >24.70 MPL for IgM aCL, >15.30 for IgG anti-β_2_GP1, and >17.00 for IgM anti-β_2_GP1 were considered positive [17].

IgG/IgM anti-Ps/Pt were determined by ELISA using a Tecan Sunrise absorption microplate spectrophotometer (Austria) with a Serin-Prothrombin-GM reagent kit for the detection of antibodies from AESKULISA. IgG/IgM aPS/Pt were measured in U/mL. On the basis of the mean values of IgG/IgM aPs/Pt in the control group, the levels of positivity were identified using the formulas: arithmetic mean (M) + 3 or 5 standard deviations (SD): M + 3SD and M + 5SD. The diagnostic value of the distinguished positivity levels and the levels proposed by the reagent manufacturers (>18.0 U/mL) was assessed [18, 19]. On the basis of the results of the analysis, the positivity levels were as follows: >73.60 U/mL (M + 5SD) for IgG aPs/Pt and >18.00 IU/mL for IgM aPs/Pt.

The study of LA was performed on an automatic coagulometer manufactured by Siemens Healthcare (Germany) using screening (LA1) and confirmatory (LA2) tests. LA was determined in patients who did not receive anticoagulants. LA was determined in 35 out of 115 patients included in the study. Due to the small number of patients for whom LA was determined, this parameter, which is estimated at 4 points, was not used in our study when calculating GAPSS.

Arterial hypertension was diagnosed at an increase in systolic blood pressure (BP) >140 mmHg and/or diastolic blood pressure >90 mmHg on the basis of the results of at least two measurements or when using oral antihypertensive drugs.

The level of total cholesterol in the blood serum was determined by the standard enzymatic procedure and was interpreted in accordance with the values obtained at the time of inclusion of patients in the study. Hyperlipidemia was registered at an increase in the level of total cholesterol and triglycerides above the reference values ( 3.90–6.20 mmol/L for total cholesterol and 0.00–2.30 mmol/L for triglycerides).

When statistically processing the results, the following indices were used to describe quantitative variables: arithmetic mean (*M*), standard deviation (δ), median, 25th and 75th percentiles. the For qualitative variables, frequency was used. Differences were considered statistically significant at *p* < 0.05. For quantitative variables, a normal distribution test was performed. For the parameters whose distribution differed from normal when comparing two groups, the Mann–Whitney test was used. To analyze the difference in frequencies in two independent groups of study subjects, χ^2^ (Pearson’s test) was used. ROC analysis was used to determine the sensitivity and specificity of GAPSS. The area under the curve (AUC) was estimated in the range of 0–1: <0.60 low, 0.61–0.80 moderate, and ≥0.81 high diagnostic accuracy [18, 19]. ROC curves were built using the SPSS Statistics 13.0 for Windows software package (IBM Corp., United States). Computations were performed on a personal computer using the Statistica 10.0 for Windows (StatSoft Inc., United States), SPSS Statistics 13.0 for Windows (IBM Corp., United States), and VassarStats software packages.

## RESULTS AND DISCUSSION

In 58 of 115 (50%) patients, a history of thrombosis  was revealed, including arterial thrombosis in 14 (24%), venous in 29 (50%), and combined in 15 (26%). Pregnancy on the background of the disease was in 43 women included in the study. 29 (67%) of them had obstetric pathology (Table 2). In patients with thrombosis and obstetric pathology, GAPSS was statistically significantly higher than in the absence of such disorders (*p* = 0.001, Table 3).

**Table 3.** GAPSS score in patients with systemic lupus erythematosus and clinical manifestations of antiphospholipid syndrome (thrombosis and/or obstetric pathology)

Patients with SLE	GAPSS	
	*M + SD*	*Range*
*In classical manifestations of antiphospholipid syndrome*		
With thrombosis and/or obstetric pathology (*n* = 68)	7.17 ± 5.64*	0.00–16.00
Without thrombosis and/or obstetric pathology (*n* = 47)	4.48 ± 4.55	0.00–13.00
*In thrombosisc*		
With thrombosis (*n* = 58)	7.31 ± 5.70**	0.00–16.00
Without thrombosis (*n* = 57)	4.00 ± 4.81	0.00–13.00
Arterial thrombosis only (*n* = 14)	10.42 ± 5.30***	0.00–16.00
Venous thrombosis only (*n* = 29)	5.82 ± 5.28	0.00–13.00
Combined thrombosis (*n* = 15)	7.26 ± 6.00	0.00–16.00
Patients with one thrombotic event (*n* = 22)	6.00 ± 5.60	0.00–16.00
Patients with recurrent thrombosis (2 or more) (*n* = 36)	7.94 ± 5.76	0.00–16.00
*In obstetric pathology*		
With obstetric pathology (*n* = 29)	6.68 ± 5.69	0.00–16.00
—Before the 10th week of gestation (*n* = 22)	6.63 ± 5.66	0.00–16.00
—At the 10th week of gestation (*n* = 15)	8.00 ± 5.90	0.00–16.00
—Preeclampsia/eclampsia or fetoplacental insufficiency (*n* = 12)	7.25 ± 5.83	0.00–13.00
Without obstetric pathology (*n* = 14)	5.50 ± 5.93	0.00–13.00

GAPSS, Global AntiphosPholipid Syndrome Score; SLE, systemic lupus erythematosus; APS, antiphospholipid syndrome; the incidence of obstetric pathology was determined in women who had a pregnancy against the background of the disease (*n* = 43); *р = 0.0003 compared to patients with SLE without thrombosis and/or obstetric pathology; **р = 0.001 compared to patients without thrombosis; ***p = 0.01 compared to venous thrombosis only.

In patients with and without obstetric pathology, the index in the GAPSS scale did not differ statistically significantly (Table 3).

To stratify patients into groups of low and high risk of recurrence of vascular complications, we used the threshold value GAPSS  ≥ 10, proposed in the development of this index by Sciascia et al. [9], and GAPSS ≥ 6 (10 minus 4, since the result of determining LA was not used in calculating GAPSS) (Table 4). At both thresholds, a statistically significant association between GAPSS and clinical manifestations of APS was found.

**Table 4.** Relationship of GAPSS to thrombosis and obstetric with obstetric pathology at thresholds ≥6 and ≥10, *n* (%)

GAPSS values		Thrombosis and/or obstetric pathology		p OR [25–75 percentile]
		yes (*n* = 68)	no (*n* = 47)	
≥ 6	*+*	35 (51)	10 (21)	0.001 4.00 [1.69–10.00]
< 6	*–*	33 (49)	37 (79)	
≥ 10	*+*	32 (47)	8 (17)	0.001 4.30 [1.78–11.11]
< 10	*–*	36 (53)	39 (83)	

GAPSS, Global AntiphosPholipid Syndrome Score; OR, odds ratio.

**Fig. 1.**
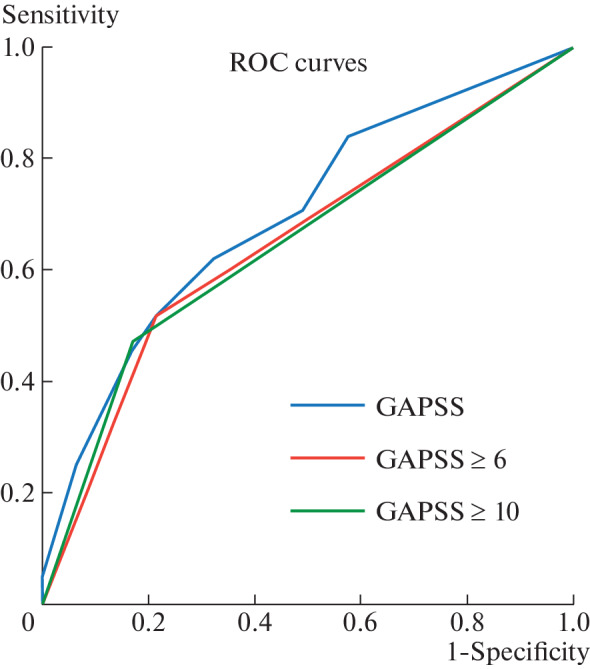
ROC curves for GAPSS as a function of thrombosis and/or obstetric pathology at thresholds  ≥6 and ≥10.

According to the results of ROC analysis, the AUC for GAPSS was 0.697 (Table 5). The ROC curves are shown in Fig. 1.

**Table 5.** Some GAPSS parameters and suggested thresholds based on ROC-analysis

Variables	AUC	p	CI		Se	Sp	PPV	NPV
			25%	75%				
GAPSS	0.697	0.0001	0.601	0.793	–	–	–	–
GAPSS ≥ 6	0.651	0.006	0.550	0.752	51	78	77	52
GAPSS ≥ 10	0.650	0.006	0.550	0.751	47	82	80	51

AUC, area under curve; CI, confidence interval; Se, sensitivity; Sp, specificity; PPV, positive predictive value; NPV, negative predictive value; GAPSS, Global AntiphosPholipid Syndrome Score.

According to the results of the ROC analysis, the GAPSS thresholds ≥6 and ≥10 had a moderate diagnostic accuracy (Table 5).

APS is a heterogeneous disease with a wide variability in clinical course and laboratory profile. The difficulty of all studies related to aPL is determined by the fluctuations in the levels of these antibodies over time from high positive to low. In some patients, after verification of APS, the result of aPL determination may become negative [2]. In addition, when APS is detected, patients are prescribed anticoagulants that prevent the development of thrombosis and obstetric pathology, and the assessment of the effect of aPL is also hampered due to therapy. We can judge the effect of aPL only at late diagnosis of the disease, when the patient has a history of recurrent thrombosis or recurrent fetal loss syndrome before prescription of anticoagulants. However, anticoagulant therapy does not always protect against vascular complications. This largely depends on the selection of anticoagulants, the patient’s adherence to treatment, as well as the development of complications caused by anticoagulant therapy. Assessment of the risk of recurrence or the development of clinical manifestations of APS largely determines the success of further therapy. Therefore, it is necessary to have a tool that allows stratification of patients into groups of high and low risk of developing thrombosis and/or obstetric pathology.

A number of clinical studies proved the clinical significance of the GAPSS index developed by Sciascia et al. [10–13, 20, 21]. Corresponding results were obtained in multivariate regression analysis. For the development of GAPSS, the significance of risk factors identified by multivariate analysis was expressed by the authors in points in proportion to the value of the corresponding β-regression coefficient (rounded to the nearest whole number) using its linear transformation. The coefficient of each variable was divided by 0.54 (lowest β value corresponding to arterial hypertension in their cohort) and rounded to the nearest whole number. The used formula was expressed as follows: point GAPPS = [β_x_/β_min_], where β_x_ is the β‑regression coefficient for the considered variable *x*, and β_min_ is the smallest β value among the significant variables after multivariate analysis. For example, in their cohort, the GAPPS score for hyperlipidemia was 3, because GAPPS = [hyperlipidemia/arterial hypertension] = [1.73/0.54] = [3.20] = 3, rounded to the nearest whole number.

In our study, an incomplete version of this scale was used, since the determination of LA in patients receiving anticoagulant therapy is impractical due to the false positive test results. It is also inappropriate to use anamnestic data, because aPL levels can change, and the risk of recurrence of clinical manifestations should be assessed at the time of the patient’s examination. Temporary withdrawal of anticoagulants for the study of LA is associated with risks of thrombosis. In some countries, heparin neutralizers and DOAC-Stop® (Direct oral anticoagulants-Stop) are used, which make it possible to study LA against the background of anticoagulant therapy and calculate GAPSS with LA. In our laboratory, heparin neutralizers and DOAC-Stop® are not used.

We evaluated GAPSS in 115 patients with SLE, not including LA in the number of initial parameters. Limitations associated with the possibility of determining aPL also affected the studies by Radin et al. [20] and Fernandez Mosteirin et al. [21]. Since aPS/Pt are not included in the APS classification criteria and their determination is not a routine test in the majority of clinical laboratories, the authors used GAPSS and excluded aPs/Pt (adjusted GAPSS, aGAPSS, variant). The authors of both studies noted that, despite the exclusion of aPs/PT, GAPSS is a valid and simple tool for stratification of the risk of thrombosis in patients with APS and/or other autoimmune diseases in daily clinical practice.

It is noteworthy that, in addition to the aPL profile, GAPSS also takes into account the conventional cardiovascular risk factors, which is consistent with the two-hit hypothesis in the APS pathogenesis. According to this theory, aPL (first hit) creates conditions for hypercoagulability, and thrombus formation is induced by additional mediators (second hit) that enhance the activation of the blood coagulation cascade already induced by aPL. The conventional cardiovascular risk factors function as additional mediators in GAPSS. Radin et al. [20] found no independent correlation between aPL positivity and cardiovascular risk factors; when determining GAPSS, both factors contribute to the development of clinical manifestations of APS. Barinotti et al. [22] assessed the cardiovascular risk in SLE patients with and without APS by comparing GAPSS (without aPS/PT, aGAPSS) and a calculator for assessing the risk of heart attack and stroke (Cardiovascular Risk, QRISK3). These authors studied 142 SLE patients aged 25 to 85 years. Of these, 34 (23.9%) had SLE with APS and 108 (76.1%) had SLE without APS. When the entire cohort was considered, the patients with cerebrovascular/coronary events had higher aGAPSS values than those without such disorders (10.10 ± 6.20 and 5.80 ± 6.10, respectively; *p* = 0.007); however, there were no significant differences in QRISK3 between them. Moreover, a significant association between the occurrence of these events and high aGAPSS risk was found (*p* = 0.03 for aGAPSS ≥ 8, *p* = 0.01 for aGAPSS ≥ 9; and *p* = 0.008 for aGAPSS ≥ 10). By focusing on the aPL profile, regardless of diagnosis, the authors found statistically significant differences in aGAPSS only between the aPL-positive and aPL-negative patients (9.60 ± 6.30 and 4.10 ± 5.10, respectively; *p* <0.001). Barinotti et al. [23] concluded that, although QRISK3 is more accurate than the conventional index in predicting cardiovascular risk in SLE patients, aGAPSS is the most valuable tool for this purpose.

GAPSS was evaluated retrospectively in 143 SLE patients with a history of pregnancies [23]. The patients with 3 or more consecutive early miscarriages (<10 weeks), fetal death >10 weeks, preterm birth (<34 weeks), preeclampsia (<34 weeks), stillbirth, and placental infarction had significantly higher GAPSS values compared with the patients without pregnancy complications. The odds ratio (OR) of having any disease during pregnancy with GAPSS = 8 was 20 compared to those with GAPSS = 1 (*p* < 0.001). Del Barrio-Longarela et al. [24] concluded that aGAPSS is not a valuable tool for revealing patients at risk of obstetric complications. The authors included in the study 137 women with aPL and a history of pregnancy, of which 65 met the API classification criteria. Sixty-one women had obstetric manifestations associated with APS and 11 were asymptomatic carriers of aPL. The risk in patients was assessed as low at aGAPSS < 6 (*n* = 73), medium at 6≤ aGAPSS ≤11 (*n* = 40), and high at aGAPSS ≥ 12 (*n* = 24). Since arterial hypertension and hypercholesterolemia in this population were rare (<10%), the aGAPSS score was determined mainly by the aPL profile. When considering patients according to aGAPSS (high, medium, and low risk), there were no significant differences in the incidence of pregnancy loss (29, 25, and 22%, respectively) or adverse pregnancy outcome (33, 47, and 33%, respectively).

When analyzing the associations of thrombosis in terms of their localization, we noted that higher GAPSS values are associated with arterial thrombosis, which can be explained by the aPL profile. The likelihood of developing arterial thrombosis is higher at triple aPL positivity, which reflects the GAPSS value.

We found no association between higher GAPSS values and recurrent thrombosis and/or obstetric pathology, which is most likely due to ongoing therapy that prevents the development of recurrent episodes of thrombosis. In addition, there were patients who, at the time of thrombosis and/or obstetric pathology, had high aPL levels and triple positivity, and at the time of inclusion in the study their aPL levels were lower and/or there was a single aPL positivity, which significantly reduced GAPSS. In addition, in the SLE + APS patients, the ongoing SLE therapy should be borne in mind, because immunosuppressants affect aPL levels.

According to Mosteirin et al. [21] and Oku et al. [12], GAPSS threshold values may differ from those proposed by Sciascia et al. [9] depending on the cohort. In a later study, Sciascia et al. [11] showed that patients with PAPS had a high risk of recurrent thrombosis at GAPSS ≥ 11. Radin et al. [20] suggested using 10 points as a threshold value, despite the absence of aPs/Pt determination. Garcia et al. [25] evaluated the results of using aGAPSS in a multicenter cohort of SLE patients in Argentina. The study included 296 patients with SLE; 121 (41%) of them had thrombosis and/or obstetric pathology. The best index for determining the risk of thrombosis and/or complications of pregnancy, according to the results obtained, was GAPSS ≥ 4. The authors performed multivariate logistic regression analysis, the results of which were consistent with this conclusion: aCLs were an independent risk factor for thrombotic events (OR = 2.1 (95% confidence interval, 1.16–3.90); *p* = 0.015).

Taking into account these data and the absence of LA in the calculation of GAPSS, we analyzed GAPSS thresholds  ≥6 and ≥10. These index values had moderate diagnostic accuracy. According to the recommendations of the European Alliance of Associations for Rheumatology (EULAR, 2019) [26], the high-risk aPL profile includes a positive result of the test for LA (determined in accordance with the requirements of the International Society for the Study of Thrombosis and Hemostasis), or double positivity for aPL (any combination of two of the three aPL, including LA, aCL, or anti-β_2_-2GP1), or triple positivity for aPL (positivity for all three types of aPL or persistently high aPL levels). Based on these recommendations and considering our results, without LA determination, it seems more appropriate to use 6 points as a threshold to reveal patients at low and high risk of recurrent vascular complications.

## CONCLUSIONS

GAPSS values ≥6 should be used to include patients with SLE in the group of high risk of recurrent vascular complications. Further prospective follow-ups are required to confirm the value of GAPSS.
